# Circulating levels of IL-18 are significantly influenced by the IL-18 +183 A/G polymorphism in coronary artery disease patients with diabetes type 2 and the metabolic syndrome: an Observational Study

**DOI:** 10.1186/1475-2840-10-110

**Published:** 2011-12-05

**Authors:** Trine B Opstad, Alf Å Pettersen, Harald Arnesen, Ingebjørg Seljeflot

**Affiliations:** 1 Center for Clinical Heart Research, Department of Cardiology, Oslo University Hospital, Ulleval, Norway; 2 Center for Heart Failure Research, Oslo University Hospital, Norway; 3 Faculty of Medicine, University of Oslo, Norway

**Keywords:** Single nucleotide polymorphisms, IL-18 mRNA, diabetes type 2, metabolic syndrome, hypertension

## Abstract

**Background:**

Increased IL-18 serum levels have been associated with diabetes type 2, metabolic syndrome and the severity of atherosclerosis. The present study investigated the presence and influence of IL-18 genetic variants on gene- and protein expression in stable coronary artery disease (CAD) patients.

**Methods:**

The +183 A/G (rs 5744292), -137 G/C (rs 187238) and -607 C/A (rs 1946518) polymorphisms were determined in 1001 patients with angiographically verified stable CAD, and in 204 healthy controls. IL-18 gene-expression was measured in circulating leukocytes in 240 randomly selected patients. Circulating IL-18 and IL-18 binding protein levels were measured immunologically in all patients.

**Results:**

The +183 G-allele associated significantly with lower serum levels of IL-18 (*p *= 0.002, adjusted for age, glucose, body mass index and gender) and a 1.13- fold higher IL-18 gene-expression (*p *= 0.010). No influence was observed for the -137 G/C and -607 C/A polymorphisms. The IL-18 binding protein levels were not influenced by IL-18 genotypes. IL-18 levels were significantly higher in men as compared to women, and in patients with diabetes type 2 and metabolic syndrome compared to those without (*p *≤ 0.001, all). The reduction in IL-18 levels according to the +183 G-allele was 3-4 fold more pronounced in diabetes and metabolic syndrome as compared to unaffected patients.

Finally, the +183 AA genotype was more frequent in patients with hypertension (*p *= 0.042, adjusted for age, body mass index and gender).

**Conclusion:**

The reduction in serum IL-18 levels across increasing numbers of +183G-alleles was especially apparent in patient with diabetes type 2 and metabolic syndrome, suggesting a beneficial GG genotype in relation to cardiovascular outcome in these patients.

**Clinical Trial Registration Number:**

ClinicalTrials.gov: NCT00222261

## Introduction

With the knowledge that atherosclerosis is influenced by an inflammatory process, IL-18 is one of the inflammatory biomarkers that lately has been in focus amongst researchers in cardiovascular disease (CVD). Being a member of the IL-1cytokine family and a pleiotropic pro-inflammatory cytokine, the molecule plays an important role in the inflammatory cascade [1]. IL-18 is also known as an interferon gamma (IFNγ) - inducing factor [2]. After being cleaved by Caspase-1, the biologically active molecule is secreted and may be neutralized by a naturally occurring high-affinity IL-18 binding protein (IL-18 BP), modulating IL-18s' interaction with the IL-18 receptor (IL-18R) [3,4]. The binding of the free unbound IL-18 molecule to the α-chain of the cell-bound heterodimer IL-18R is required for signal transduction, mediated by the β-chain [5]. The downstream activation enhances the maturation of T-cells and natural killer cells and the production of cytokines, chemokines, cell-adhesion molecules, IFNγ and matrix metalloproteinases (MMPs), among other effects [1,6]. Both IL-18 and its receptor are expressed in active macrophages, endothelial cells and vascular smooth muscle cells and increased IL-18 expression has also been demonstrated in atherosclerotic plaques [7,8].

Elevated circulating levels of Il-18 have been shown associated with atherosclerotic lesions, diabetes type 2 (T2DM), metabolic syndrome (MetS), hypertension (HT), and a worse prognosis in coronary artery disease (CAD), although with conflicting results [9-13].

The levels of IL18 are influenced by many factors of which genetic polymorphisms may contribute. Recent studies have shown that some genetic variants of IL-18 may influence the risk and prognosis of CVD as well as modifying the IL-18 expression and circulating levels of IL-18, although the available genetic data suffers from disparity [14-18].

To further extend the knowledge and the importance of IL-18 gene variants, we aimed in the present study to investigate the frequencies of the 3'untranslated region (UTR) +183 A/G and the promoter -137 G/C and -607 C/A IL 18 polymorphisms in a large population of stable CAD. Their influence on IL-18 levels, and gene expression in circulating leukocytes as well as their distribution in subgroups of patients were assessed. To explore IL-18 activity over its endogenous antagonist, levels of IL-18 BP were evaluated accordingly in the same cohort. Our hypotheses were that the genetic polymorphisms would translate into variable levels of IL-18 and possibly also IL-18 BP, and further be differently distributed in subgroups of CAD.

We have in the present study demonstrated a significant association between G- allele of the +183 A/G polymorphism and lower circulating IL-18 levels, which specifically was present in patients with T2DM and MetS. The frequency of the +183 AA genotype was higher in CAD patients with HT, but without any relation to CAD itself.

## Methods

### Study population

A total of 1001 patients (97% Caucasians) with stable CAD, all angiographically verified, enrolled in the ASCET trial [19] and 204 healthy individuals (mean age 55 years, 28% females, all Caucasians) were studied for the selected IL-18 polymorphisms. The healthy individuals were included after an interview, clinical examination, and a near-maximum exercise bicycle electrocardiogram to rule out any clinical evidence for cardiovascular disease. Circulating IL-18 and IL-18 BP levels were measured in all patients and IL-18 gene-expression was analyzed in a cohort of 240 randomly selected patients in the CAD group.

The study was approved by the Regional Ethics Committee and all patients gave their written informed consent to participate. The ASCET study is registered at the website; clinicaltrials.gov, identification number: NCT00222261.

### Clinical subgroups

Within the CAD population, previous myocardial infarction (MI) was recorded by patients' medical files and HT was defined as previous diagnosed and treated HT. Diabetes included individuals with treated T2DM and/or fasting glucose > 7.0 mmol/L and MetS was defined according to the American Heart Association/National Heart, Lung and Blood Institute [20]. Smoking habits were recorded as current smokers or not. Ex-smokers were included as non-smokers if they had quit smoking ≥ 3 months ago.

### Blood collection

In fasting conditions between 8.00-10.00 a.m., blood samples were collected and prepared for routine analyses (lipids, glucose, HbA1c) by use of convential methods, and for determination of IL-18 and IL-18 BP along with DNA and RNA extraction.

### Genotyping

DNA was extracted from EDTA whole-blood by using the MagNA Pure LC DNA Isolation kit on the MagNA Pure LC Instrument (Roche Diagnostics, Germany). Genotyping of the promoter polymorphisms -137 G/C (rs187238) and -607C/A (rs1946518) were performed using allele specific primers and probes included in the TaqMan^® ^SNP Genotyping Assays; the C_11655953_10, C_ 2898460_10, respectively (Applied Biosystems, Foster City, CA, USA). The genotyping of the 3'UTR +183A/G polymorphism (rs5744292) was performed using a Custom Design Assay manufactured by Applied Biosystems' The Custom TaqMan^® ^Assays Service. Allelic discriminations for all three polymorphisms were performed by the ABI Prism 7900 HT Sequence Detection system along with the TaqMan^® ^Genotyping Master Mix (Applied Biosystems). About 5% of the samples were repeated, with 100% concordance. For the +183 A/G SNP assay, samples with verified genotype were included, kindly provided by Dr. Laurence Tirét, Faculté de Médicine, Paris, France.

### Gene-expression

Total RNA was extracted from PAXgene^® ^Blood RNA tubes, by using the PAXgene^® ^Blood RNA Kit (PreAnalytix, Qiagen GmbH, Germany), including an extra cleaning step (RNeasy^®^MinElute^® ^Cleanup Kit, Qiagen). Total RNA (range 200-800 ng/μL) was reversely transcribed in a total volume of 20 μl, using the Omniscript^® ^RT Kit (Qiagen), Oligos (dTs) and Rnase Inhibitor (Applied Biosystems). IL-18 mRNA levels were determined by real-time PCR on the ViiA™ 7 Real Time PCR System (Applied Biosystems), including the TaqMan^® ^Gene Expression Assay (HS00155517_m1) and TaqMan^® ^Fast Universal PCR Master Mix (2X) No AmpErase^® ^UNG (Applied Biosystems). The IL-18 mRNA levels were normalized to β-2-microglobulin (HS99999907_m1, Applied Biosystems) and fold expression (relative quantification using the ΔΔCt method) was determined in relation to a reference sample, as previously described [21].

### Measurements of IL-18 and IL-18 BP

Serum concentrations of IL-18 and IL-18 BP were determined using the Human IL-18 ELISA kit (Medical Biological Laboratories, Naka-ku Nagoya, Japan) and the Human IL-18 BP ELISA kit (R&D Systems Europe, Abingdon, Oxon, UK), the latter measuring isoenzyme *a *of the IL-18 BP. Whether the IL-18 assay measures the free IL-18 molecule and/or IL-18 bound to IL-18 BP cannot be clarified by the manufacturer. The intra- and inter-assay coefficients of variation for IL-18 were 2.1% and 8.1%, respectively, and for IL-18 BP, 8.8% and 4.2%.

### Statistics

The Chi square test was used to test for deviation from Hardy-Weinberg equilibrium (HWE). Group comparisons were performed by Kruskal-Wallis test and Mann-Whitney test when appropriate for continuous data and Chi square test for categorical data. For correlation analysis, Spearman's Rho was applied. A logistic regression model was used to adjust for potential covariates in the association between genotypes and clinical subgroups, i.e. if significant in univariate comparisons. To adjust for relevant covariates in the association between genotypes and phenotypes, a linear regression model was used. A two-tailed probability test of 0.05 or less was considered statistically significant. Exact p-values are given, except when p > 0.2. All statistical analyses were performed by SPSS 16.0 (SPSS Inc).

## Results

Characteristics of the CAD population are shown in Table 1. All patients were on aspirin, 20% were diagnosed with T2DM and 24% with MetS, 44% had previously suffered MI, 56% were treated for HT, and 22% were women. The +183 A/G and -607 C/A polymorphisms were successfully analyzed in 996 individuals and the -137 G/C in 995, and further in all controls (n = 204).

**Table 1:** Characteristics of the CAD population (n = 1001).

Age (years, mean (range))	62 (36-81)
Men/Women n (%)	783/218 (78/22)
Type 2 Diabetes Mellitus n (%)	200 (20)
Myocardial infarction n (%)	436 (44)
Hypertension n (%)	553 (56)
Metabolic syndrome n (%)	244 (24)
SBP (mmHg)	139.4 (19.3)
DBP (mmHg)	82.1 (9.7)
Current smokers n (%)	204 (20.4)
BMI (kg/m^2^)	27.9 (11.5)
Total cholesterol (mmol/L)	4.6 (1.0)
HDL cholesterol (mmol/L)	1.3 (0.4)
LDL cholesterol (mmol/L)	2.5 (0.8)
Triglycerides (mmol/L)	1.6 (1.1)
Fasting glucose (mmol/L)	6.0 (1.9)
IL-18 pg/mL	248.0 (195.3, 322.7)*
IL-18 BP pg/mL	23.2 (19.2, 28.2)*
HbA1c (%)	6.0 (0.9)
Medication %	

Statins	98
Aspirin	100
β-Blockers	76
Nitrates	22
ACE inhibitors	26

Values are mean (SD) or number (proportions) if not otherwise stated

SBP: systolic blood pressure; DBP: diastolic blood pressure; BMI: body mass index; ACE: angiotensin converting enzyme.

* Median levels (25- and 75 percentiles)

### Frequencies of IL-18 polymorphisms

The obtained frequencies of the +183 A/G, -137 G/C and -607 C/A polymorphisms are in line with previous reports and no deviation from HWE was observed, except for deviation in controls for the +183 A/G polymorphism. As shown in Table 2, frequencies of the polymorphisms were not differently distributed in CAD patients and controls. We observed two stable genotype combinations in both cohorts; the +183 GG genotype was always associated with wild-types of the two promoter polymorphisms, whereas the -137 CC and -607 AA genotypes were always connected with the +183 wild-type (AA).

**Table 2:** Frequencies of the IL-18 polymorphisms in CAD patients and controls

IL-18 Genotype		CAD (%)	Controls (%)	*p*-value *
+183 A/G	AA	543 (55)	114 (56)	
	AG	391 (39)	85 (42)	
	GG	62 (6)	5 (2)	
G-allele frequency		0.259	0.234	> 0.2

-137 G/C	GG	532 (53)	108 (53)	
	GC	394 (40)	82 (40)	
	CC	69 (7)	14 (7)	
C-allele frequency		0.267	0.270	> 0.2

-607 C/A	CC	364 (37)	74 (36)	
	CA	500 (50)	96 (47)	
	AA	132 (13)	34 (17)	
A-allele frequency		0.384	0.403	> 0.2

* Mann-Whitney test, heterozygous and homozygous of the minor allele pooled

### Serum IL-18 and IL-18 BP concentrations in relation to IL-18 genotypes

**Figure 1:**
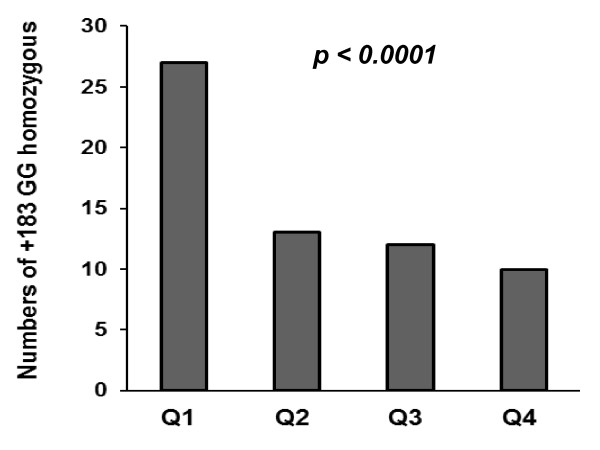
**Numbers of +183 homozygous (GG) according to quartiles (Q) of IL-18 (pg/mL): **Q1 < 195, Q2 = 196-248, Q3 = 249-323, Q4 > 324.

The +183 A/G polymorphism associated significantly with IL-18 levels in the total CAD population, with lower levels across increasing numbers of G-alleles (*p *< 0.0001), still significant after adjustments for age, glucose, body mass index (BMI) and gender (*p *= 0.002) (Table 3). When dividing IL-18 levels into quartiles, significantly higher numbers of homozygous patients (GG) in the lowest quartile was observed (*p *< 0.0001, for trend) (Figure 1). IL-18 levels did not differ according to the -137 G/C and -607 C/A genotypes in the total CAD population. However, in men alone, the -607 A-allele associated weakly with higher IL-18 levels as compared to the -607 CC genotype (*p *= 0.053) (medians (25, 75 percentiles): 258 (207, 336) versus 246 (196, 313) pg/mL), respectively, however, not statistically significant when adjusted for covariates (*p *> 0.2). IL-18 BP levels were significantly correlated to IL-18 levels (r = 0.31, *p *< 0.0001). No differences in IL-18 BP levels according to any of the investigated genotypes were observed.

**Table 3:** Serum Levels of IL-18 in relation to the IL-18 polymorphism in the total CAD population

Genotype		Il 18 pg/mL*	*p*-value †
+183	AA	260 (204, 336)	***<*0.0001**^#^
	AG	236 (193, 310)	
	GG	211 (155, 284)	

-137	GG	248 (198, 323)	> 0.2
	GC	245 (193, 316)	
	CC	270 (192, 368)	

-607	CC	243 (190, 313)	> 0.2
	CA	249 (199, 322)	
	AA	251 (192, 360)	

* Median levels (25- and 75 percentiles)

† Kruskal-Wallis test

^# ^*p *= 0.002 after adjustments for age, glucose, BMI and gender

### IL-18 gene-expression

The clinical characteristics of the subpopulation in which gene-expression was performed (n = 240), were similar to that of the total CAD group, except for a higher frequency of patients with T2DM in the subpopulation (28% versus 20%).

The +183 G-allele induced a 1.13 fold increase in the levels of IL-18 mRNA in circulating leukocytes (*p *= 0.010), whereas none of the promoter polymorphisms influenced IL-18 gene-expression. The gene-expression data did not differ between subgroups of CAD.

Neither circulating IL-18 levels nor the IL-18 BP levels correlated to expression levels of the IL-18 gene (r = -0.083, *p *= 0.2 *and *r = 0.15, *p *= 0.8, respectively).

### Frequencies of IL18 polymorphisms in subgroups of CAD

**Figure 2:**
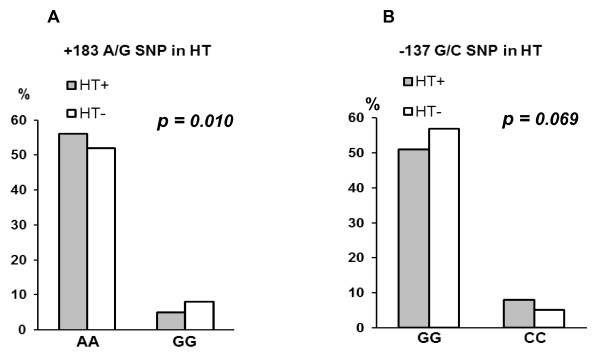
**The presence of HT according to IL-18 gene variants: **The percentage of HT in homozygous individuals of major and minor alleles of the +183 A/G polymorphism (A) and the -137 G/C polymorphism (B), versus non-HT patients. P-values are adjusted for age, body mass index and gender. As the -607 AA genotype always is present when the -137 CC genotype exist, only the -137 CC genotype is presented in Figure 2B.

When comparing the presence of HT in relation to the +183 AA, AG and GG genotypes, a significant higher frequency of the AA genotype was observed in HT-patients (p = 0.034), still significant after adjustment for age, BMI, and gender (p = 0.042). The +183 homozygous mutant (GG) individuals were simultaneously wild-types of both the -137 (GG) and -607 (CC) polymorphisms, and the homozygous of the -137 and -607 variant alleles in parallel (CC and AA, respectively) were persistently wild-types (AA) of the +183 polymorphism. An assessment of the homozygous mutants versus wild types of the polymorphisms revealed a reduced risk for having HT when being +183 GG homozygous with an OR of 0.50 (9 5% CI 0.29, 0.85, *p *= 0.009), still significant after adjustments for covariates (*p *= 0.010) (Figure 2A). Furthermore, an increased risk for having HT was related to the presence of the -137 CC and -607 AA genotypes combined, with an OR of 1.66 (95% CI 0.98, 2.8, *p *= 0.055), however less prominent after adjustment for the covariates (*p *= 0.069) (Figure 2B).

No other associations between the investigated polymorphisms and subgroups of CAD, i.e. T2DM, MetS, previous MI and gender, were observed.

### Serum IL-18 and IL-18 BP concentrations in CAD subgroups as related to IL-18 genotypes

In the total CAD population, significantly higher levels of IL-18 were found in patients with T2DM and MetS as compared to patients without (*p *≤ 0.001, both). Additionally, higher IL-18 levels were observed in men as compared to women (*p *< 0.0001) (Table 4.) No difference in IL-18 levels was observed in the previous MI and HT subgroups and between smokers and non-smokers. Further, in patients with MetS, the IL-18 BP levels were slightly, but significantly elevated (*p = *0.010).

**Table 4:** Serum levels of IL-18 and IL-18 BP in relation to CAD subgroups

Disease Entities		IL 18 pg/mL*	*p*-value †	IL 18 BP pg/mL*	*p*-value †
MI	**+**	252 (197, 327)	> 0.2	23.5 (19.4, 28.5)	0.191
	**-**	245 (194, 320)		23.0 (19.2, 28.2)	

HT	**+**	248 (198, 318)	> 0.2	23.5 (19.5, 29.1)	0.066
	**-**	248 (190, 327)		22.8 (18.9, 27.2)	

T2DM	**+**	259 (207, 364)	**0.001**	23.6 (19.6, 29.0)	0.163
	**-**	244 (192, 311)		23.0 (19.2, 28.1)	

MetS	**+**	265 (217, 356)	**< 0.0001**	24.1 (19.8, 29.3)	**0.010**
	**-**	243 (188, 310)		22.8 (19.1, 28.0)	

Men		256 (203, 329)	**< 0.0001**	23.2 (19.3, 28.3)	> 0.2
Women		221 (176, 290)		23.1 (18.9, 28.1)	

Current Smokers	**+**	252 (207, 323)	> 0.2	23.0 (19.2, 27.1)	> 0.2
	**-**	247 (194, 323)		23.2 (19.3, 28.7)	

* Median levels (25- and 75 percentiles)

† Mann-Whitney test

**Figure 3:**
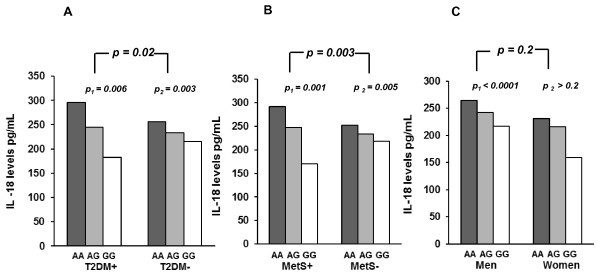
**Effects of the +183 A/G polymorphism on IL-18 circulating levels in different CAD subgroups: **Differences in the G-alleles' lowering effects in patients with T2DM (A) and Mets (B) as compared to unaffected patients, and in men as compared to women (C). p_1 _and p_2 _values refer to trend analyses across genotypes. p-values refer to differences in changes between groups across genotypes.

Due to the strong association between the +183 A/G polymorphism and circulating IL-18 levels (Table 3), analysis of IL-18 levels in the subgroups (T2DM, Mets, and gender) according to the +183 A/G polymorphism were performed. The G-allele associated significantly with lower IL-18 levels in patients both with and without T2DM (*p *= 0.006 and *p *= 0.003, respectively) (Figure 3A), and in patients with and without MetS (*p = *0.001 and *p = *0.005, respectively) (Figure 3B). However, in diabetics versus non-diabetics a significantly more pronounced reduction in IL-18 levels in +183 GG-subjects compared to AA subjects was observed (*p *= 0.02) (Figure 3A). In MetS patients versus non-MetS patients this difference was even more pronounced (*p *= 0.003) (Figure 3B). The reduction in IL-18 levels across +183 genotypes was significantly present in men (*p *< 0.0001), whereas no difference in the reduction between gender was observed (*p *= 0.2) (Figure 3C).

## Discussion

The main findings in the present study were the significant association between the G-allele of the IL-18 +183 A/G polymorphism and lower circulating IL-18 levels, which specifically was present in patients with T2DM and MetS. The frequency of the +183 AA genotype was higher in CAD patients with HT, but without any relation to CAD itself.

The +183 G-alleles' influence on IL-18 levels shown in our relatively large study of stable CAD patients is in accordance with results from one previous study in a similar population [15]. The polymorphisms' functional mechanism has not been clarified. As the location of the +183 A/G polymorphism is in the 3'UTR of the gene, an interference with mRNA stability or the translational process is suggested, as well as an interaction with the 5' end in regulating the transcriptional activity. Although no difference in allelic degradation of mRNA and no difference in expression of an upstream reporter gene were found in functional experiments [17], these possibilities cannot be excluded. The +183 A/G polymorphism is reported to be in complete linkage disequilibrium (LD) with the 5'UTR -105C/T polymorphism, both being parts of 6 major haplotypes defined by 5 polymorphisms in the IL-18 gene [15]. It was shown that the haplotype carrying the +183 G-allele was the one associated with the lowest IL-18 levels [15], supporting the polymorphisms' functional role in lowering IL-18 levels.

The +183 polymorphism modified slightly the expression of the IL-18 gene in circulating leukocytes, with higher mRNA levels related to the G-allele. This result seem to present a discrepancy to the lower IL-18 circulating levels, and also to previous gene-expression results from lymphoblastoid cell-lines [17]. The observed 1.13-fold increase may not be of biological significance. Further, circulating leukocytes may not be the main source contributing to circulating IL-18, although IL-18 mRNA was shown to be constitutively expressed in human peripheral blood mononuclear cells due to lack of a destabilizing sequence in the 3'UTR of the IL-18 gene [22]. A post-translational impact of the inflammasome and Caspase-1, in the cleavage of pro IL-18 to its active secreted form, may contribute to the inconsistency. The results may, nevertheless, also indicate a compensatory reaction from the circulating inflammatory cells.

We observed no influence of the -137 G/C and -607 C/A polymorphisms on circulating IL-18 levels or on IL-18 gene expression. These two promoter polymorphisms have previously been associated with lower transcriptional activity in *in vitro *studies [18,23,24]. However, in a human population no association was observed for the -137 G/C variant [23].

Significantly higher IL-18 levels were found in male patients as compared to females. This gender difference is to our knowledge novel information. In a recent meta-analysis, in which nine of twelve studies included both gender, the association between IL-18 and CVD events was generalized to both gender [25]. However, an increased IL-18 related risk of CV events in men warrant further investigation. Interestingly, an experimental study performed in mice indicated that increased endogenous IL-18 production reduced survival only in the male animals [26].

Increased IL-18 levels in patients withT2DM and MetS have previously been reported, and our observations support these results [9,11,27-29]. The importance of IL-18 in MetS was also underlined in a review by Trøseid et al. [30]. It was also recently shown increased IL-18 mRNA expression in adipose tissue from MetS subjects compared to non-MetS individuals [31]. We could further demonstrate that the influence of the +183 A/G polymorphism was especially apparent in the T2DM and Mets groups, suggesting a beneficial influence of the G-allele in these patients. G-allele carriers have also been reported to have reduced frequency of MetS and the subsequent IL-18 levels were suggested to be of genetic origin [32]. Although the frequency of the +183 G-allele was not differently distributed in MetS and T2DM patients as compared to the unaffected patients in the present study, an association is still feasible.

The observed risk of having HT was significantly affected by the presence of the combined genotypes of the three polymorphisms investigated (+183 A/G, -137 G/C and -607 C/A). The +183 GG genotype (in combination with -137 GG and -607 CC) was associated with a 50% lower HT risk, whereas the +183 AA, in combination with -137 CC and -607 AA, was associated with a 70% higher risk. It should be emphasized that our stable CAD patients with HT did not present higher IL-18 levels, which might be due to their treatment regimen. Nevertheless, as the combined genotypes in the total population were associated with lower and higher circulating IL-18 levels, respectively, a related mechanism is plausible.

An inflammatory state accompanied by the A-allele of the +183 polymorphism may consequently induce a cumulative increased risk for HT. Genotypes are fixed during life-time and a pro-inflammatory state initiated early in life may consequently lead to development of HT over time in these genotype-specific patients. We have previously shown that arterial stiffness was associated with both elevated levels of IL-18 and elevated systolic blood pressure in MetS patients [33]. Chronic elevated levels of IL-18 may lead to a persistently increased expression of IL-18 inducible cytokines downstream of the NF-κB/AP-1 signal pathway, i.e. IFNγ and MMPs [34,35], which are of importance for an inflammatory state in atherosclerosis in general and for arterial remodeling, important in HT. HT has on the other hand been shown to induce an upregulation of IL-18 through β2-adrenergic receptor stimulation in endothelial cells. Thus, a positive feed-back mechanism has been suggested [36].

In the attempt to sort out the role of IL-18 BP in regulating IL-18 levels, our results were not clarifying. The levels of IL-18 and IL-18 BP were significantly correlated, and as the expression of IL-18 BP has been shown to be markedly up-regulated by IFNγ, a negative feedback mechanism on IL-18 activity has been suggested [37]. The interrelated values may also be a consequence of the methodology used in measuring the two markers. The results indicate that the utilized IL-18 assay also measures IL-18 in complex with IL-18 BP and not only the free IL-18 molecule, also stated by others [38,39]. As the manufacturer cannot clarify what the assay exactly measures, no valid conclusion can be drawn.

### Limitations

The control group is small and seven years younger than the patient population, which may limit the statistical value of these results. The controls were, however, only included in the gene-analysis. As the +183 A/G polymorphism deviates from HWE in controls, probably due to small sample size, the observed related frequencies should be carefully interpreted.

In summary, the +183 A/G polymorphism induced lower IL-18 levels consistently and significantly, and has conceivably a functional role in regulating the transcriptional and/or the translational process. The beneficial +183 GG genotype was especially apparent in relation to IL-18 levels in MetS and T2DM patients. The modified risk for HT according to the different genotypes indicates an importance of this gene in HT patients, possibly through IL-18 levels. As HT, MetS and T2DM are associated with worse prognosis in CVD, the regulatory mechanisms of IL-18, especially through the +183 A/G polymorphism should be further explored.

## Abbreviations

ASCET: aspirin and clopidogrel end-point trial; BMI: body mass index; CAD: coronary artery disease; CVD: cardiovascular disease; HT: hypertension; HWE: Hardy-Weinberg equilibrium; IL-18 BP: IL-18 binding protein; IFNγ: interferon gamma; LD: linkage disequilibrium; MetS: metabolic syndrome; MI: myocardial infarction; MMP: matrix metalloproteinase; OR: odd ratio; PCR: polymerase chain reaction; SNP: single nucleotide polymorphism; T2DM: type 2 diabetes mellitus; UTR: untranslated region.

## Conflict of interests

The authors declare that they have no competing interests.

## Authors' contributions

TBO conducted the study and was responsible for laboratory and statistically analysis and drafting the manuscript. AÅP contributed to the study protocol, acquired data and contributed in discussion of the manuscript. HA contributed to the study protocol, the interpretation of results and discussion of the manuscript. IS conducted the study, contributed to the interpretation of results, and discussion of the manuscript. All authors have read and approved the manuscript.
